# Altered β_1−3_-adrenoceptor influence on α_2_-adrenoceptor-mediated control of catecholamine release and vascular tension in hypertensive rats

**DOI:** 10.3389/fphys.2015.00120

**Published:** 2015-04-20

**Authors:** Torill Berg

**Affiliations:** Division of Physiology, Department of Molecular Medicine, Institute of Basic Medical Sciences, University of OsloOslo, Norway

**Keywords:** α_2_-adrenoceptors, β-adrenoceptors, hypertension, total peripheral vascular resistance, sympathetic nervous system, catecholamine release, spontaneously hypertensive rats

## Abstract

α_2_- and β-adrenoceptors (AR) reciprocally control catecholamine release and vascular tension. Disorders in these functions are present in spontaneously hypertensive rats (SHR). The present study tested if α_2_AR dysfunctions resulted from altered α_2_AR/βAR interaction. Blood pressure (BP) was recorded through a femoral artery catheter and cardiac output by an ascending aorta flow probe. Total peripheral vascular resistance (TPR) was calculated. Norepinephrine release was stimulated by a 15-min tyramine-infusion, which allows presynaptic release-control to be reflected as differences in overflow to plasma. Surgical stress activated some secretion of epinephrine. L-659,066 (α_2_AR-antagonist) enhanced norepinephrine overflow in normotensive controls (WKY) but not SHR. Nadolol (β_1+2_) and ICI-118551 (β_2_), but not atenolol (β_1_) or SR59230A [β_(3)/1*L*_] prevented this increase. All βAR antagonists allowed L-659,066 to augment tyramine-induced norepinephrine overflow in SHR and epinephrine secretion in both strains. Inhibition of cAMP-degradation with milrinone and β_3_AR agonist (BRL37344) enhanced the effect of L-659,066 on release of both catecholamines in SHR and epinephrine in WKY. β_1/2_AR antagonists and BRL37344 opposed the L-659,066-dependent elimination of the TPR-response to tyramine in WKY. α_2_AR/βAR antagonists had little influence on the TPR-response in SHR. Milrinone potentiated the L-659,066-dependent reduction of the TPR-response to tyramine. Conclusions: β_2_AR activity was a required substrate for α_2_AR auto inhibition of norepinephrine release in WKY. β_1+2_AR opposed α_2_AR inhibition of norepinephrine release in SHR and epinephrine secretion in both strains. βAR-α_2_AR reciprocal control of vascular tension was absent in SHR. Selective agonist provoked β_3_AR-G_i_ signaling and influenced the tyramine-induced TPR-response in WKY and catecholamine release in SHR.

## Introduction

**Figure 1:**
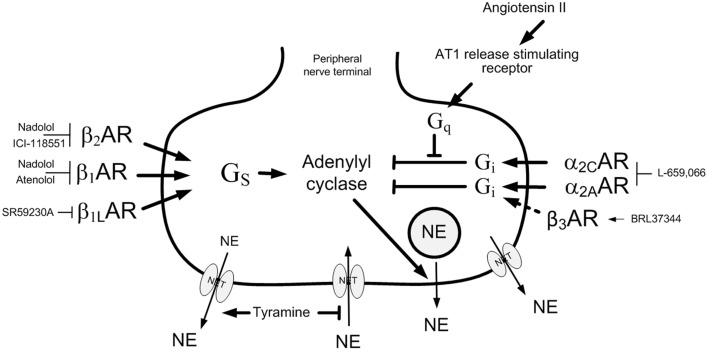
**Control of norepinephrine release from peripheral sympathetic nerve endings**. Tyramine stimulates norepinephrine release by reverse transport through NET. Consequently, re-uptake through NET is prevented, and presynaptic modulation of vesicular release is reflected as differences in overflow to plasma (Berg et al., 2012; Berg and Jensen, 2013). The release of norepinephrine from secretory granules is activated by adenylyl cyclase, which is stimulated by β_1,1L,2_AR-G_s_ and inhibited by α_2_AR-G_i_. In WKY, α_2_AR auto inhibition required β_2_AR activity, but was independent of β_1_AR signaling. In SHR, blocking β_1,1L,2_AR activity allowed α_2_AR inhibition of release. The β_3_AR-selective agonist BRL37344 reduced norepinephrine overflow in SHR but not WKY (dotted arrow). The β_3_AR antagonist SR59230A reduced overflow apparently due to its abililty to inhibit β_1L_AR and not the β_3_AR. The action of antagonists and agonist are indicated. NE, norepinephrine; Pointed arrows, postive effects; Blunted arrows, inhibitory actions.

α_2_- and β-adrenoceptors (AR) comprise the α_2A, B,and C_ and the β_1,2,and 3_ subtypes. By coupling to inhibitory (G_i_) and stimulatory (G_s_) G-proteins, respectively, α_2A,B,C_- and β_1+2_AR have opposite effect on adenylyl cyclase, and therefore have reciprocal actions on various functions involved in the control of blood pressure (BP), including the release of catecholamines and vascular tension. The β_3_AR has been shown to couple to G_i_ and induce a negative inotropic effect in the heart (Gauthier et al., 1998). Transmitter release from peripheral sympathetic nerves is inhibited by presynaptic α_2_AR, primarily the α_2A_-subtype (Trendelenburg et al., 2003; Berg and Jensen, 2013), and stimulated by presynaptic β_2_AR (Stjarne and Brundin, 1976; Westfall et al., 1979). Also the β_1_AR has recently been demonstrated to enhance norepinephrine release, providing a plausible rational for the antihypertensive action of β_1_AR antagonists, the most frequently used β-blockers in the treatment of hypertension (Berg, 2014a). α_2_AR auto-receptors in addition inhibited adrenal epinephrine secretion in *in vivo* experiments (Brede et al., 2003; Berg et al., 2012), whereas β_1_- or β_2_AR antagonists had no effect (Berg, 2014a). α_2_AR-mediated auto inhibition of neuronal and adrenal catecholamine release has been shown to be dysfunctional in the spontaneously hypertensive rat (SHR) (Berg and Jensen, 2013). This dysfunction may contribute to the hyper adrenergic and hypertensive state in this model of human hypertensive disease, in agreement with the high plasma norepinephrine concentration and hypertension observed in α_2A_AR-gene-deleted mice (Makaritsis et al., 1999). The failing α_2_AR auto inhibition in SHR may result from an altered interaction between different presynaptic receptors, as indicated by the restored α_2_AR function in SHR after α_2C_AR stimulation or angiotensin AT1 receptor inhibition (Berg, 2013) (Figure 1). The β_3_AR has been shown to be less sensitive to catecholamine-induced desensitization than the β_1_- and β_2_AR (Mallem et al., 2004; Rouget et al., 2004), and a β_3_AR up-regulated and β_1_AR down-regulated relaxation was demonstrated in SHR thoracic aortic rings (Mallem et al., 2004). It may therefore be hypothesized that alterations in βAR signaling may alter α_2_AR auto inhibition of catecholamine release in SHR.

**Figure 2:**
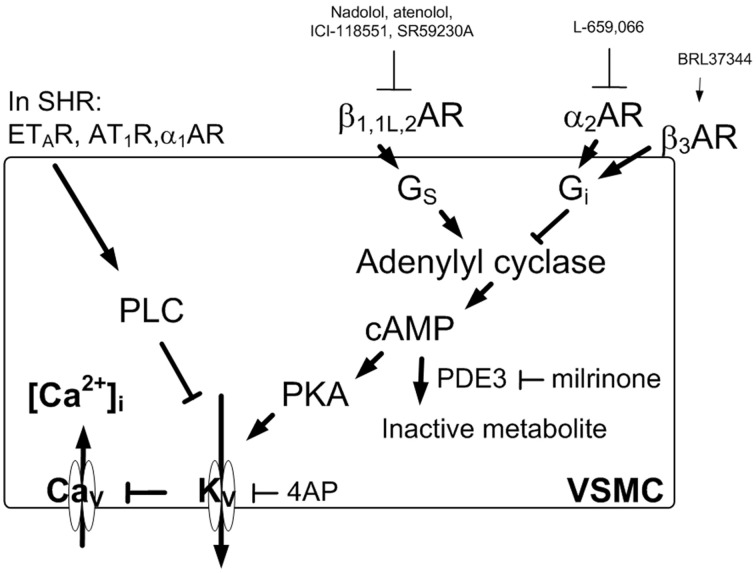
**AR-mediated control of tension in VSMC**. Inhibition of K_V_ induces depolarization which will activate Ca^2+^ influx through Ca_v_ and thus precipitates vasoconstriction due to a rise in [Ca^2+^]_i_. K_V_ is stimulated by cAMP-PKA signaling, and in pathophysiologic conditions such as that in SHR may be inhibited by PLC. The BRL37344-induced β_3_AR-G_i_ signaling and increased TPR was only observed in WKY during tyramine-induced norepinephrine release. Ca_v_, voltage sensitive Ca^2+^ channels; K_V_, voltage sensitive K^+^ channels; 4AP, the K_V_ inhibitor 4-aminopyridine; PKA, protein kinase A; Arrow, positive action; Blunted arrow, negative action.

α_2B_AR (Philipp et al., 2002) and βAR are also present in vascular smooth muscle cells (VSMC), where they modulate the α_1_AR-mediated vasoconstrictory response to norepinephrine (Figure 2). VSMC tension is in addition influenced by endothelial α_2A_AR and β_2_AR, which both stimulate nitric oxide (NO) synthesis (Shafaroudi et al., 2005; Queen et al., 2006). Also vasodilatory and vasoconstrictory α_2_AR-mediated control of total peripheral vascular resistance (TPR) appeared dysfunctional in SHR (Berg and Jensen, 2011, 2013).

Presynaptic receptors modulate norepinephrine release from the nerve terminal vesicles. This control is not reflected by differences in norepinephrine overflow to plasma, due to that the response is terminated by re-uptake through the norepinephrine re-uptake transporter (NET). Presynaptic control of release is therefore not easily studied *in vivo*. However, tyramine stimulates norepinephrine release by reversing the transport through NET (Figure 1), and consequently prevents re-uptake through NET. Presynaptic modulation of concomitant vesicular release can therefore be demonstrated as differences in overflow to plasma (Berg et al., 2012; Berg and Jensen, 2013). The presynaptic receptors will be stimulated by the released norepinephrine and/or by other agonists present in the vicinity. Secreted epinephrine is not subjected to re-uptake through NET, and was therefore not influenced by tyramine, but was stimulated to some extent by the stress induced by the experiment itself (Berg et al., 2012). Thus, the inhibitory effect of presynaptic α_2_AR on catecholamine release could be demonstrated by the ability of the non-selective, peripherally restricted, α_2_AR antagonist L-659,066 to increase tyramine-induced norepinephrine overflow and epinephrine secretion (Berg and Jensen, 2013). The stimulating effect of presynaptic βAR was demonstrated by pre-treatment with β_1or2_AR antagonists (Berg, 2014a). Inhibition of release could be stimulated in SHR by the β_3_AR agonist BRL37344, compatible with stimulation of β_3_AR-G_i_ activation (Berg, 2014b). The reduced release observed after the β_3_AR antagonist SR59230A was likely to result from its inhibitory effect on the G_s_-coupled low-affinity state β_1_AR (β_1L_AR) (Berg, 2014b). The use of tyramine also allowed a concomitant examination of the role of postsynaptic α_2_AR and βAR in the cardiovascular response to the released norepinephrine (Figure 2).

The purpose of the present investigation was therefore to gain a better understanding of the reason underlying the failing α_2_AR auto-inhibition of catecholamine release in SHR, i.e., to test if there was a difference in the interaction between the α_2_AR and the three βAR subtypes in this strain compared to that in their normotensive controls (WKY). A second goal was to test if a difference in β_1−3_AR activity was responsible for the failing α_2_AR influence on the TPR-response to the tyramine-stimulated norepinephrine release in SHR.

## Materials and methods

### Experimental procedure

All experiments were approved by The Norwegian Animal Research Authority (NARA) and conducted in accordance with the Directive 2010/63/EU of the European Parliament. Male, 12–14 weeks old SHR (Okamoto, SHR/NHsd strain, 273 ± 2 g body weight, *n* = 109) and their normotensive control, i.e., WKY (Wistar Kyoto, 279 ± 9 g body weight, *n* = 124) on conventional rat chow diet (0.7% NaCl) were anesthetized with sodium pentobarbital (65–70 mg/kg, IP) and tracheotomized. A heparinized catheter was inserted into the femoral artery to record systolic (SBP) and diastolic (DBP) BP. The rats were subsequently connected to a positive-pressure respirator and ventilated with air throughout the experiment. Cardiac output (CO, i.e., minus cardiac flow) and heart rate (HR) were recorded by a flow probe on the ascending aorta, connected to a T206 Ultrasonic Transit-Time Flowmeter (Transonic Systems Inc., Ithaca, NY, USA). After surgery was completed, the arterial catheter was flushed with 0.15 ml heparinized (1000 U/ml) phosphate-buffered saline (PBS; 0.01 M Na-phosphate, pH 7.4, 0.14 M NaCl). Mean arterial BP [MBP = (SBP-DBP/3) + DBP] and TPR (=MBP/CO) were calculated. Body temperature was maintained at 37−38°C by external heating, guided by of a thermo sensor inserted inguinally into the abdominal cavity. Drugs were dissolved in PBS and administered as bolus injections (0.6–1 ml/kg) through a catheter in the femoral vein, unless otherwise indicated.

### Experimental design

After a control period of about 10 min, control rats were pre-treated with vehicle (PBS), and subsequently infused with tyramine for 15 min (1.26 μmol/kg/min) (Berg, 2005; Berg and Jensen, 2013). In the experimental groups, the rats were pre-treated with drugs to modify presynaptic and postsynaptic α_2_AR or βAR signaling, separately or combined.

#### Inhibition of βAR-signaling

Inhibition of βAR-signaling was achieved by βAR-antagonists, the peripherally restricted, i.e., which does not cross the blood-brain barrier, nadolol (β_1+2_, 8.5 μmol/kg), or atenolol (β_1_, 5.6 μmol/kg), the not-restricted ICI-118551 (β_2_, 1 μmol/kg initial dose, then 0.3 μmol/kg/min throughout the experiment) or the β_3_AR antagonist SR59230A (13.8 μmol/kg) (Berg et al., 2010). SR59230A also inhibited the putative β_4_AR (Malinowska and Schlicker, 1997), later identified as β_1L_AR (Kaumann et al., 2001).

#### Amplification of βAR-signaling

Amplification of βAR-signaling was achieved by pre-treatment with the phosphodiesterase 3 (PDE3) inhibitor milrinone (1.4 μmol) (Berg et al., 2009) which will prevent degradation of cyclic AMP (cAMP) (Figure 2). The β_3_AR was stimulated by the agonist BRL37344 ((±)-(R^*^,R^*^)-[4-[2-[[2-(3-Chlorophenyl)-2-hydroxyethyl]amino]propyl]phenoxy]-acetic acid sodium hydrate) infused at a rate of 1 nmol/kg/min throughout the experiment (Malinowska and Schlicker, 1997; Berg, 2014b).

#### Inhibition of α_2_AR-signaling

Inhibition of α_2_AR-signaling was achieved by pre-treatment with the none-selective, peripherally restricted α_2_AR antagonist L-659,066 (Clineschmidt et al., 1988) (4.4 μmol/kg) (Berg et al., 2012; Berg and Jensen, 2013).

#### Inhibition of G_i_ signaling

Since G_i_ represents a main signaling pathway for all α_2_AR, G_i_-signaling was abolished by the G_i_-inhibitor *Bordetella pertussis* toxin (PTX, 15 μg/kg, i.p., −48 h) (Anand-Srivastava et al., 1987). The latter rats were pre-treated with PBS during the experiment.

#### The interaction between α_2_AR and βAR signaling

The interaction between α_2_AR and βAR signaling was studied by combining βAR antagonists/agonist/milrinone with the α_2_AR antagonist L-659,066 as indicated in Table 1. Ten min was allowed between drugs, except for SR59230A which was followed by L-659,066 or tyramine after 5 min.

**Table 1:** **Cardiovascular baselines after pre-treatment, i.e., prior to tyramine and, in parenthesis, the response to pre-treatment**.

**Pre-treatment**	**WKY**				**SHR**			
	**MBP**	**HR**	**CO**	**TPR**	**MBP**	**HR**	**CO**	**TPR**
	**mm Hg**	**beats/min**	**ml/min**	**mm Hg/ml/min**	**mm Hg**	**beats/min**	**ml/min**	**mm Hg/ml/min**
PBS (control)^b^	66±3	341±8	32±2	2.1±0.1	92±5^*^	391±5^*^	19±1^*^	4.6±0.2^*^
	(−3±2)	(−6±4)	(2±0)	(−0.3±0.1)	(−5±5)	(−16±5)	(0±1)	(−0.3±0.2)
**INHIBITION OF βAR SIGNALING**								
Nadolol (β_1+2_ANT)^b^	65±2	338±5	31±2	2.1±0.1	64±5^†^	345±10^†^	13±2^†^	4.6±0.5
	(−3±1)	(−21±6)	(1±1)	(−0.2±0.0)	(−25±2)^†^	(−80±7)^†^	(−1±1)	(−2.0±0.8)^†^
Atenolol (β_1_ANT)^b^	61±2	338±9	28±2	2.4±0.1	63±3^†^	338±9	13±1^†^	5.1±0.4
	(−12±1)	(−32±7)^†^	(1±1)	(−0.5±0.1)	(−32±5)^†^	(−69±10)^†^	(−4±1)	(−0.4±0.5)
ICI-118551 (β_2_ANT)^b^	50±4	311±12	28±4	1.8±0.1	70±4	358±7^†^	18±1	4.3±0.2
	(−6±2)	(−31±4)^†^	(2±1)	(−0.4±0.1)	(−23±4)	(−59±8) ^†^	(1±1)	(−1.2±0.2)
SR59230A (β_3_+β_1L_ ANT)^b^	84±4^†^	390±8^†^	27±2	3.3±0.3^†^	90±8	424±13	18±2	5.1±0.4
	(10±3)^†^	(30±7)^†^	(4±1)	(−0.2±0.2)	(−6±5)	(−21±5)	(0±1)	(−0.5±0.3)
**STIMULATION OF THE βAR SIGNALING PATHWAY**								
Milrinone (PDE3 INH)	40±2^†^	343±7	30±2	1.4±0.1^†^	52±3^†^	413±14	18±1	2.9±0.2^†^
	(−35±1)^†^	(2±8)	(−2±1)	(−1.0±0.1)^†^	(−59±7)^†^	(−1±9)	(−4±1)	(−2.4±0.5)^†^
BRL44408 (β_3_AGON)^b^	51±2^†^	312±9	44±4	1.2±0.1^†^	109±7	409±15	23±1^†^	4.8±0.3
	(3±3)	(−8±9)	(9±2)^†^	(−0.6±0.1)^†^	(−9±7)	(0±9)	(4±1)^†^	(−0.4±0.3)
**INHIBITION OF α_2_AR-G_i_ SIGNALING**								
PBS after PTX (G_i_ INH)^a^	52±3	338±9	39±4	1.3±0.2^†^	65±5	389±12	22±3	3.3±0.5
	(−2±2)	(−21±4)	(3±1)	(−0.1±0.1)	(−8±6)	(−24±11)	(−1±2)	(−0.5±0.2)
L-659,066 (α_2_ANT)^b^	59±7	345±11	35±2	1.7±0.1	69±6^†^	398±12	17±1	4.3±0.4
	(−17±2)^†^	(−10±5)	(2±0)	(−0.6±0.0)^†^	(−21±4)^†^	(−16±10)	(−1±0)	(−1.1±0.2)
**INTERACTION BETWEEN βAR AND α_2_AR SIGNALING**								
Nadolol + L-659,066	47±6^‡^	323±8	23±3	2.0±0.1	61±4^†^	356±8^†^	15±2	4.4±0.4
	(−6±2)†	(−30±10)	(3±2)	(−0.7±0.2)	(−18±7)	(−69±9)^†^^⊣^	(−1±1)	(−0.8±0.6)
Atenolol + L-659,066	56±5	340±10	27±4	2.2±0.2	67±5	363±8	14±1	4.7±0.3
	(−18±5)^†^	(−52±18)^†^	(5±2)	(−1.1±0.3)^†^	(−35±4)^†^^⊣^	(−90±7)^†^^⊣^	(0±1)	(−2.3±0.2)^†^^‡⊣^
ICI-118551 + L-659,066	49±4	336±7	26±1	1.9±0.2	77±7	356±4^†^	19±1	4.0±0.1^†^
	(−26±3)^†^^‡^	(−39±7)^†^^⊣^	(1±1)	(−1.2±0.1)^†^^⊣^	(−16±9)	(−68±1)^†^^⊣^	(0±1)	(−1.1±0.2)
SR59230A + L-659,066	55±6^‡^	364±25	28±3	2.0±0.2^‡^	88±9	408±11	19±2	4.7±0.4
	(−3±4)^‡^	(23±9)^⊣^	(2±3)	(−0.3±0.2)	(−3±9)	(2±9)	(−1±1)	(0.2±0.4)
Milrinone + L-659,066	37±1^†^	366±12	28±2	1.3±0.1^†^^⊣^	39±2^†^^‡⊣^	431±9^†^	19±1	2.2±0.2^†^^⊣^
	(−42±6)^†^	(−5±5)	(1±2)	(−1.5±0.2)^†^	(−62±6)^†^^⊣^	(0±15)	(−2±1)	(−3.0±0.4)^†^^⊣^
BRL44408 + L-659,066	41±4^†^	379±11^‡^	45±8	1.0±0.1^†^	77±8^‡^	407±5	25±1^†^^⊣^	3.1±0.2^†^^‡^
	(−28±6)^†^^⊣^	(20±9)	(10±7)	(−1.0±0.1)^†^^‡⊣^	(−33±9)^†^^‡^	(2±6)	(4±2)	(−2.4±0.5)^†^^‡⊣^

a Since PTX was given 4 h prior to the experiment, its effect was reflected as differences in the baselines themselves. Comparisons were made between WKY and SHR controls (^*^ after SHR-values) and between the PBS-controls and the experimental groups, using the response to PBS to evaluate the effect of pre-treatment (^†^). Comparisons were also made between groups pre-treated with βAR-antagonist + α_2_AR-antagonist and corresponding groups pre-treated with βAR-antagonist alone (^‡^) or α_2_AR-antagonist alone (⊣).

b *The rats in these groups were in part or in full the same as in (Berg and Jensen, 2013; Berg, 2014a,b). Six to ten rats were included in each group, except for the WKY and SHR controls and the SHR ICI-118551 and L-659,066 groups which comprised 17, 18, 13, and 13 rats, respectively*.

* , P ≤ 0.05;

† , P ≤ 0.0036;

‡, ⊣ *, P ≤ 0.0083*.

### Measurement of plasma catecholamines

1.5 ml blood was collected from the femoral artery into tubes containing 45 μl 0.2 M glutathione and 0.2 M ethylene glycol-bis(2-aminoethylether)-N,N,N′,N′-tetraacetic acid (EGTA) (4°C). Plasma was stored at −80°C until catecholamine concentrations were determined using 400 μl plasma and the 5000 Reagent kit for HPLC analysis of catecholamines in plasma from chromsystems GmbH, Munich, Germany, as described by the manufacturer. The samples were run on a Shimadzu monoamines analyzer system, using an isocratic flow rate of 0.8 ml/min, and an electrochemical detector (Decade II) and a SenCell electrochemical flow cell (Antec Leyden, Zoeterwoude, The Netherlands).

### Drugs

L-659,066 was a kind gift from Merck, Sharp and Dohme Labs, Rahway, NJ. ICI-118551 was obtained from ICI-Pharma, Cheshire, UK; SR59230A from Santa Cruz Biotechnology, Heidelberg, Germany; and pentobarbital from The Norwegian National Hospital, Oslo, Norway. The remaining drugs were from Sigma Chemical Co., St. Louis, MO, USA.

### Statistical analyses

Results are presented as mean values ± s.e.m. Effect of pre-treatment, differences in the cardiovascular baselines, (data averaged every min), and the plasma catecholamine concentrations were evaluated by overall tests (One-Way ANOVA), followed by two-tailed two-sample Student's *t*-tests. The cardiovascular response-curves to tyramine (data averaged every min) were analyzed using Repeated Measures Analyses of Variance and Covariance, first as over-all tests, subsequently for each group separately or between two groups. Significant responses and groups differences were subsequently located at specific times using two-tailed one- and two-sample Student's *t*-tests, respectively. For non-parametric data, two-sample Student's *t*-tests were substituted by Kruskal–Wallis tests. At each step, testing proceeded only when the presence of significant differences and/or interactions was indicated. For the cardiovascular data, the *P*-value was for all tests and each step adjusted according to Bonferroni, whereas *P* ≤ 0.05 was considered significant for the catecholamine data.

In those cases, where comparisons were made with rats included in previous publications (Table 1), these experiments were performed, in part or in full, intermittently with the present groups. When this was not the case, some of the previous experiments were substituted with new to ensure that all experiments overlapped in time. Control rats were included randomly throughout the study.

## Results

### The influence of β_1−3_AR signaling on α_2_AR-mediated inhibition of tyramine-induced norepinephrine overflow to plasma

#### The effect of modulating βAR signaling

**Figure 3:**
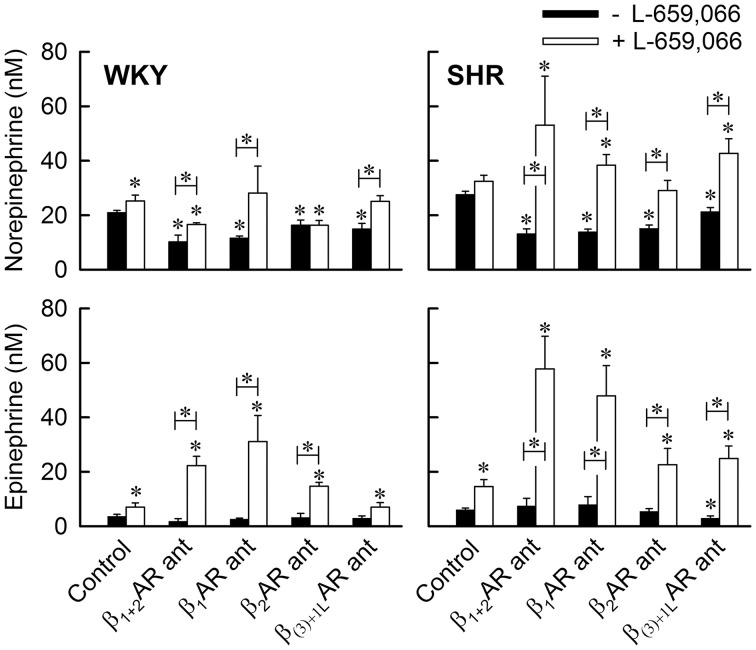
**The effect of βAR antagonist, alone or combined with the α_2_AR antagonist L-659,066, on the plasma concentration of norepinephrine and epinephrine**. Blood was sampled immediately after the 15-min tyramine-observation period, but without discontinuing the tyramine-infusion. Significant differences between the control groups pre-treated with PBS and the experimental groups (^*^ above columns), and between the corresponding groups pre-treated with βAR antagonist and βAR antagonist + L-659,066 (^*^ in bar) were located as indicated. The βAR antagonist (ant) used is indicated below columns; β_1+2_AR ant = nadolol, β_1_AR ant = atenolol, β_2_AR ant = ICI-118551, and β_(3)+1L_AR ant = SR59230A which preferably acts as an antagonist to the G_s_-coupled low-affinity state β_1_AR (Berg, 2014b). ^*^ *P* ≤ 0.05.

**Figure 4:**
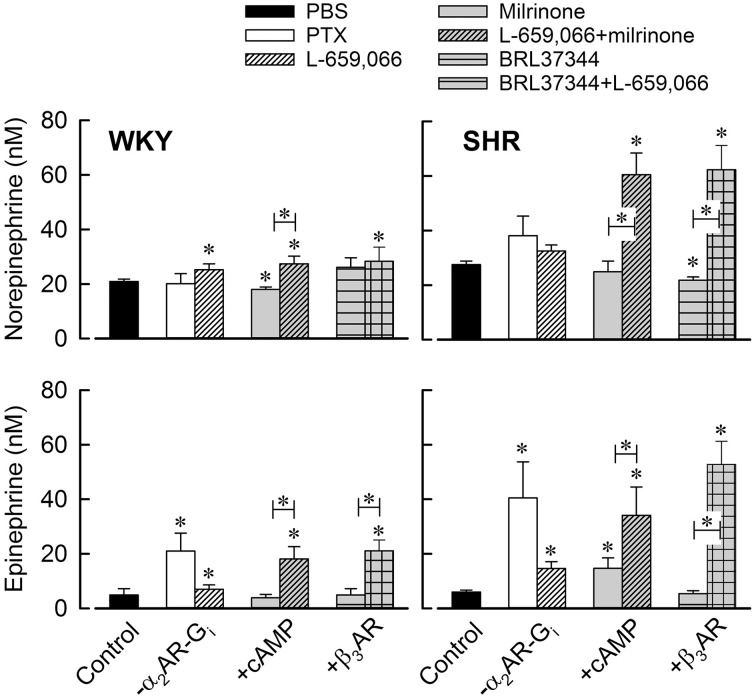
**The effect of G_i_- α_2_AR inhibition, increased cAMP or β_3_AR signaling on the plasma concentration of norepinephrine and epinephrine**. Total G_i_ inhibition was achieved by PTX and α_2_AR inhibition by L-659,066. Increased cAMP signaling was done by preventing its degradation using the PDE3 inhibitor milrinone. BRL37344 was used to stimulate β_3_AR signaling. Significant differences between the control groups pre-treated with PBS and the experimental groups (^*^ above columns), and between corresponding groups pre-treated with milrinone or β_3_AR agonist without and with additional pre-treatment with L-659,066 (^*^ in bar) were located as indicated. ^*^ *P* ≤ 0.05.

As previously described (Berg and Jensen, 2013; Berg, 2014a), all βAR antagonists, including SR59230A, reduced the tyramine-induced overflow of norepinephrine to plasma in both strains (Figure 3). Since the effect of SR59230A was likely to involve inhibition of β_IL_AR rather than β_3_AR (Berg, 2014b), this result demonstrated that the overflow was enhanced by β_1+1L+2_AR activation. However, the β_3_AR agonist BRL37344 precipitated a minor reduction in overflow in SHR but not WKY (Figure 4), compatible with a G_i_-mediated inhibition of release in SHR (Berg, 2014b).

#### The effect of inhibiting α_2_AR-G_i_ signaling

The G_i_ inhibitor PTX had no significant effect on the tyramine-induced norepinephrine overflow in either strain (Figure 4). As before (Berg and Jensen, 2013), the α_2_AR antagonist L-659,066 increased norepinephrine overflow in WKY, but the rise was not statistically significant in SHR (Figures 3, 4).

#### The interaction between α_2_AR and βAR signaling

L-659,066 eliminated the reduction in the tyramine-induced overflow induced by the β_1_AR antagonist atenolol in WKY (*P* = NS compared to the controls or L-659,066 alone, *P* = 0.004 compared to atenolol alone) (Figure 3). L-659,066 also totally reversed the reduction following pre-treatment with the β_(3)+IL_AR antagonist SR59230A (*P* = 0.005 compared to SR59230A alone, *P* = NS compared to the controls and L-659,066 alone) (Figure 3). However, L-659,066 counter-acted only in part the reduction following the β_1+2_AR antagonist nadolol (*P* ≤ 0.004 compared to the WKY controls and L-659,066 alone, *P* = 0.049 compared to nadolol alone), and did not at all counter-act the reduction induced by the β_2_-selective antagonist ICI-118551 (*P* = 0.05 and 0.005 compared to the controls and L-659,066 alone, respectively, *P* = NS compared to ICI-118551 alone) (Figure 3). These results demonstrated that α_2_AR antagonist increased tyramine-stimulated norepinephrine release in WKY also in the presence of β_1_AR and β_(3)+1L_AR antagonist, but not when the β_2_AR subtype was blocked.

The plasma norepinephrine concentration in L-659,066 + milrinone-treated WKY was higher than that in the controls (*P* = 0.05) but not different from that after L-659,066 alone (Figure 4). This result showed that enhanced cAMP signaling, i.e., down-stream of adenylyl cyclase (Figure 2), did not influence the response to L-659,066 in this strain. BRL37344 did not alter the effect of L-659,066 on tyramine-induced norepinephrine release (Figure 4), demonstrating that β_3_AR-G_i_ signaling did not contribute to the response in WKY.

In SHR, L-659,066 eliminated the reduction in tyramine-induced norepinephrine overflow induced by all four βAR antagonists (*P* ≤ 0.003 compared to βAR-antagonist alone), and was higher than that in the controls in all groups (*P* ≤ 0.018), except in the ICI-118551 + L-659,066-treated group (*P* = NS) (Figure 3). These results demonstrated that α_2_AR-mediated auto inhibition of norepinephrine release was opposed by both β_1+1L_AR and β_2_AR activity in SHR, with the β_1_AR having the greatest impact. Norepinephrine overflow was also increased after pre-treatment with L-659,066 + milrinone (*P* ≤ 0.001 compared to the control, milrinone- or L-659,066-only groups), showing that α_2_AR auto inhibition was enhanced by inhibition of cAMP degradation. In addition, the effect of L-659,066 on overflow was higher when combined with the β_3_AR agonist BRL37344 (*P* ≤ 0.006 compared to the controls and after BRL37344 or L-659,066 alone) (Figure 4).

### The effect of PTX, β_1−3_AR antagonists, milrinone, and β_3_AR agonist on α_2_AR-mediated inhibition of epinephrine secretion

#### The effect of modulating βAR signaling

The βAR antagonists (Figure 3), milrinone and β_3_AR agonist (Figure 4) had by themselves no effect on the concentration of epinephrine in plasma collected at the end of the experiment in either strain, except for a minor reduction after SR59230A (Figure 3) and an increase after milrinone (Figure 4) in SHR (*P* ≤ 0.04).

#### The effect of inhibiting α_2_AR-G_i_ signaling (Figure 4)

PTX clearly increased the secretion of epinephrine in both strains (*P* ≤ 0.032), demonstrating a tonic G_i_-mediated inhibition of epinephrine release. Some increase in the plasma epinephrine concentration was observed in L-659,066-pre-treated WKY (*P* = 0.048) and, in this collection of animals, also in the L-659,066-pre-treated SHR (*P* = 0.004). This observation demonstrated that part of the tonic inhibition of release was due to α_2_AR activity.

#### The interaction between α_2_AR and βAR signaling

However, when L-659,066 was combined with βAR-antagonist (Figure 3), the plasma epinephrine concentration was for all groups in both strains higher than that in the corresponding controls or after each drug alone (*P* ≤ 0.05). These results demonstrated that βAR signaling interfered with α_2_AR auto inhibition of epinephrine secretion. The only exceptions were the WKY SR59230A + L-659,066-pre-treated group where the plasma concentration was not different from that in the controls or after each antagonist alone, and the SHR ICI-118551 + L-659,066 group, which was not different from that after L-659066 alone (Figure 3). A potentiated effect of L-659,066 was also seen when L-659,066 was combined with milrinone or the β_3_AR-agonist BRL37344 (Figure 4).

### The role of β_1−3_AR and α_2_AR in the control of cardiovascular baselines (Table 1)

As previously documented (Berg et al., 2010; Berg, 2014a), nadolol, atenolol, and ICI-118551 reduced HR in WKY, and MBP, HR and TPR baselines in SHR, although the difference was not statistically significant for all. SR59230A increased MBP and HR in WKY but had no effect in SHR (Berg, 2014b). Milrinone alone had no significant effect on baseline HR or CO, but reduced MBP and TPR in both strains (*P* ≤ 0.003 compared to the controls). BRL37344 itself reduced TPR baseline in WKY and increased CO baseline in both strains (Berg, 2014b). Baselines in rats pre-treated with the G_i_ inhibitor PTX were not significantly different from that in the controls (*P* ≥ 0.0036), except for a reduced TPR in WKY. L-659,066 reduced baseline MBP in both strains and TPR significantly in WKY only.

After nadolol/atenolol/ICI-118551 + L-659,066, changes in the cardiovascular baselines in WKY were largely the same as the combined effect of that observed after the βAR and α_2_AR antagonists alone, except for a greater fall in TPR after ICI-118551 + L-659,066 (*P* ≤ 0.005 compared to that after each antagonist alone). In SHR, TPR baseline was reduced after atenolol + L-659,066 (*P* ≤ 0.008 compared to that after each antagonist alone), whereas the reduction following nadolol alone was not observed after nadolol + L-659,066. The TPR-response to milrinone + L-659,066 was not different from that after milrinone alone in either strain, but was greater than that after L-659,066 alone in SHR (*P* ≤ 0.005). After BRL37344 + L-659,066, the changes seen after BRL37344 or L-659,066 alone remained, but in WKY there was a further reduction in TPR (*P* ≤ 0.008 compared to BRL37344 or L-659,066 alone). L-659,066 had no effect on HR or CO baselines in either strain, and did not alter the HR-response to βAR antagonist, milrinone or β_3_AR agonist.

### The influence of β_1−3_AR and α_2_AR on the TPR-response to tyramine-stimulated norepinephrine release

**Figure 5:**
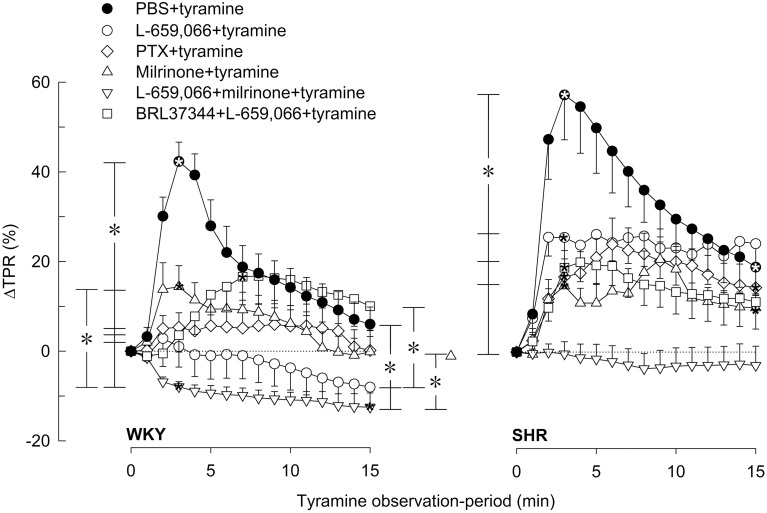
**βAR and α_2_AR influence on the TPR-response to tyramine-induced norepinephrine release in WKY and SHR**. The rats were pre-treated as indicated by symbol legends. Significant responses (^*^ within symbol) and differences between groups at peak-response (brackets left of curves) and at 15 min (brackets right of curves) were located as indicated. Comparisons were made between the control and the experimental groups, and between corresponding groups pre-treated with L-659,066 and milrinone + L-659,066. Baselines prior to tyramine are shown in Table 1. ^*^P ≤ 0.025 after curve evaluation (please see Materials and Methods for details).

**Figure 6:**
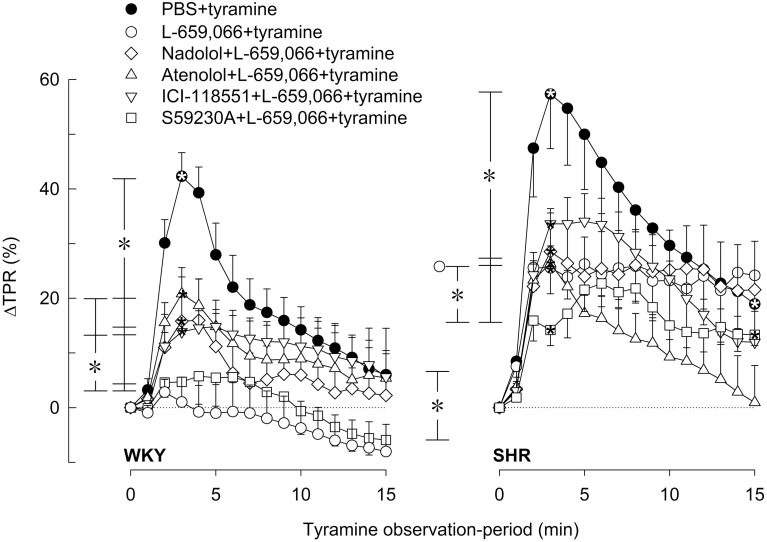
**The effect of βAR antagonists on the α_2_AR-influence on the TPR-response to tyramine-induced norepinephrine release in WKY and SHR**. The rats were pre-treated with the peripherally restricted α_2_AR antagonist L-659,066, alone or combined with βAR antagonist, as indicated by symbol legends. Significant responses (^*^ within symbol) and differences between groups at peak-response (brackets left of curves) and at 15 min (brackets right of curves) were located as indicated. Comparisons were made between the control and the experimental groups, and between corresponding L-659,066 and βAR antagonist + L-659,066 groups. Baselines prior to tyramine are shown in Table 1. *P* ≤ 0.025 after curve evaluation (please see Materials and Methods for details).

**Figure 7:**
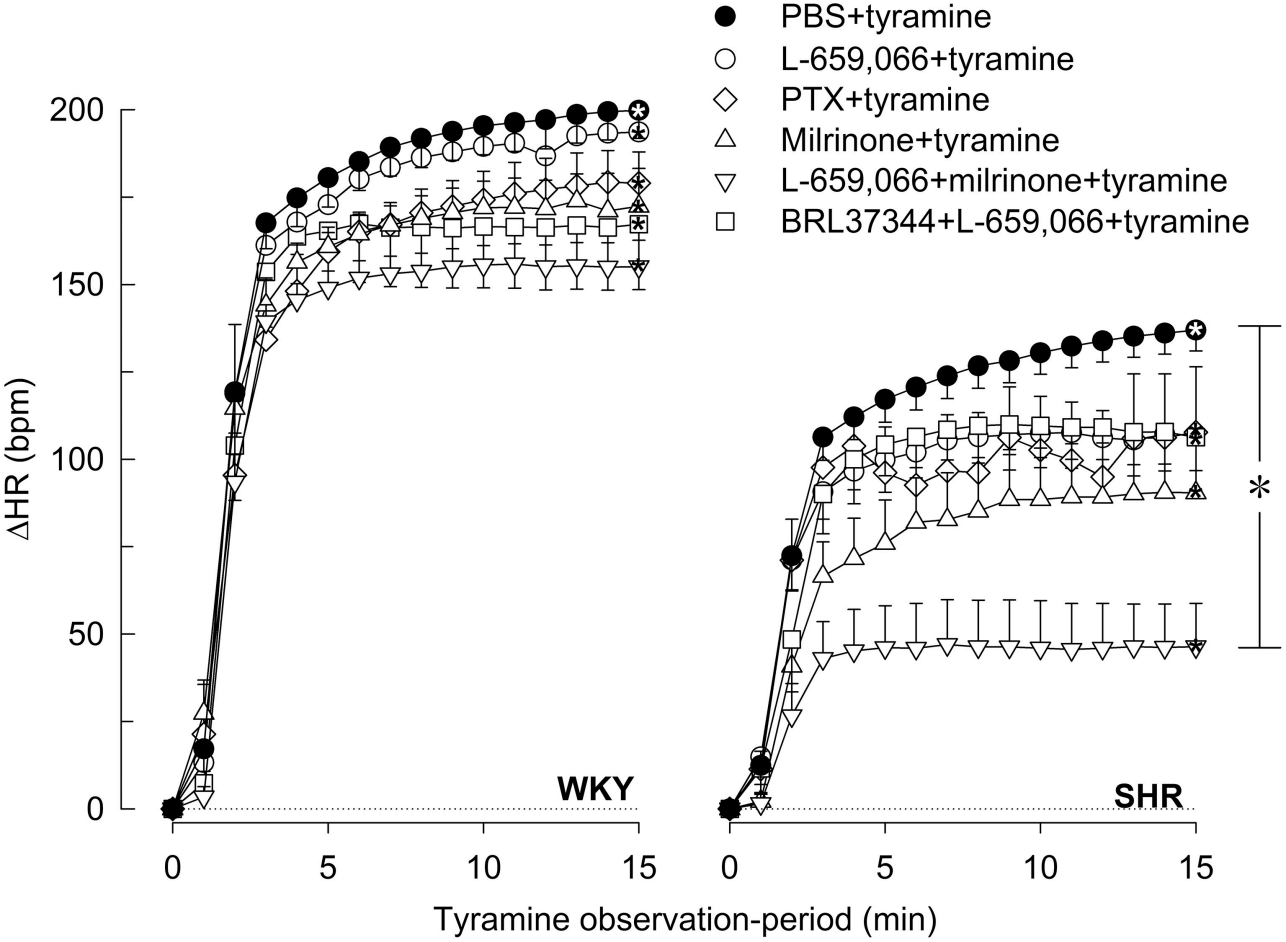
**βAR and α_2_AR influence on the HR-response to tyramine-induced norepinephrine release in WKY and SHR**. The rats were pre-treated as indicated by symbol legends. Significant differences between groups after 15 min (brackets right of curves) were located as indicated. Comparisons were made between the control and the experimental groups, and between corresponding groups pre-treated with L-659,066 and milrinone + L-659,066. Baselines prior to tyramine are shown in Table 1. ^*^P ≤ 0.05 after curve evaluation (please see Materials and Methods for details).

**Figure 8:**
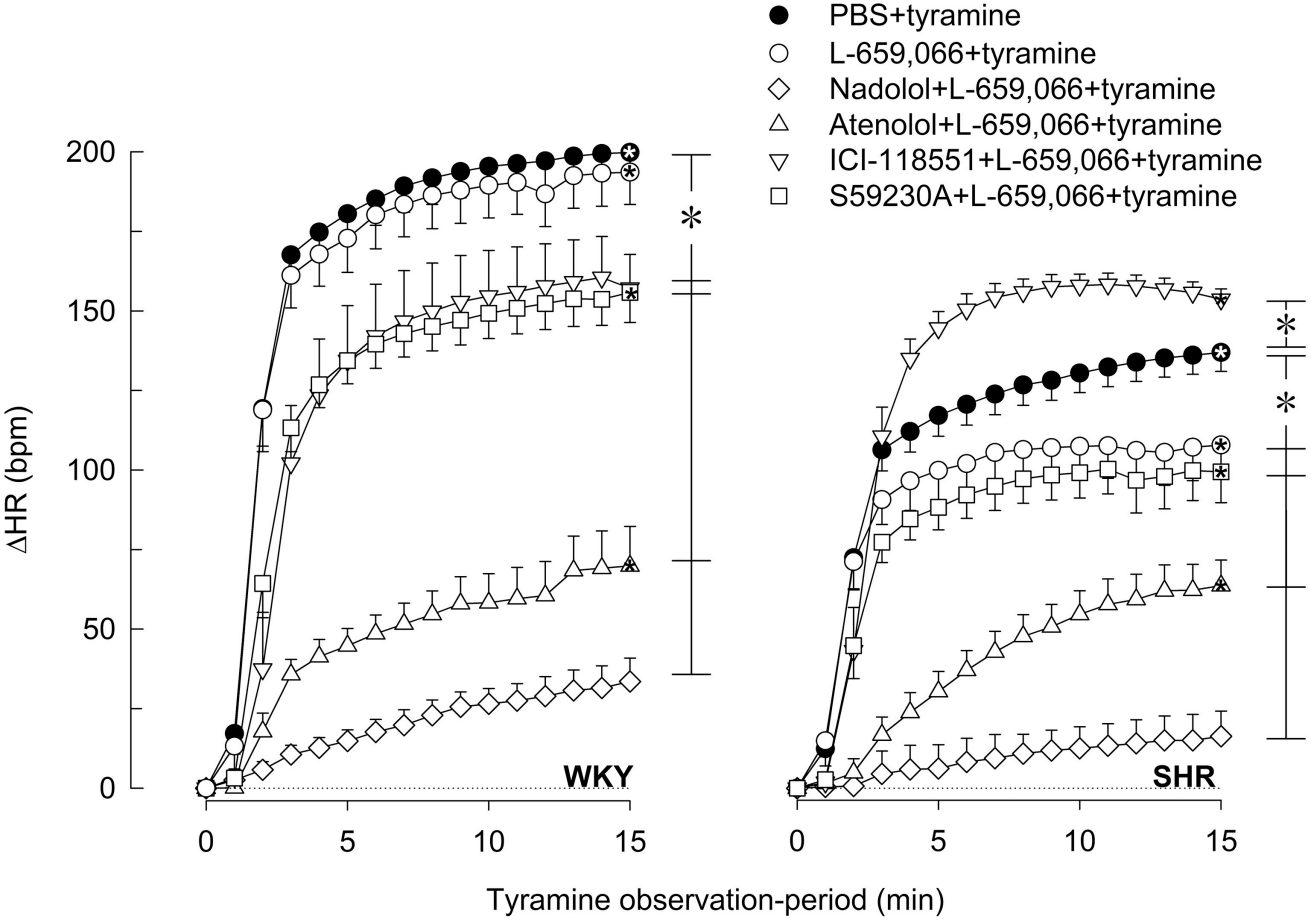
**The effect of α_2_AR-antagonist on the βAR-influence on the HR-response to tyramine-induced norepinephrine release in WKY and SHR**. The rats were pre-treated with the peripherally restricted α_2_AR antagonist L-659,066, alone or combined with βAR antagonist, as indicated by symbol legends. Significant responses (^*^ within symbol) and differences between groups after 15 min (brackets right of curves) were located as indicated. Comparisons were made between the control and the experimental groups, and between corresponding L-659,066 and βAR antagonist + L-659,066 groups. Baselines prior to tyramine are shown in Table 1. ^*^P ≤ 0.05 after curve evaluation (please see Materials and Methods for details).

In agreement with previous studies (Berg et al., 2010; Berg and Jensen, 2013), the tyramine-induced release of norepinephrine activated a transient rise in TPR (Figures 5, 6) and a sustained increase in HR (Figures 7, 8), MBP and CO (not shown) in both strains. Pre-treatment with PTX or L-659,066 eliminated the TPR-response to tyramine in WKY (*P* ≤ 0.001 compared to WKY controls, *P* = NS for single curve evaluation), and in SHR reduced the TPR-peak response (*P* ≤ 0.008), but had no effect on the later response (Figure 5). Milrinone reduced the TPR-peak response in both strains (Figure 5). After pre-treatment with milrinone + L-659,066, the tyramine-induced vasoconstriction was reversed to a vasodilatory response in WKY (*P* ≤ 0.025 compared to the control and milrinone-only groups at 3 and 15 min), and eliminated in SHR (*P* = NS, single curve evaluation) (Figure 5). When the β_3_AR agonist BRL37344 was given prior to L-659,066, the TPR-response to tyramine was higher than that after L-659,066 alone in WKY (*P* = 0.024 at 15 min) but not different in SHR (Figure 5).

The L-659,066-dependent elimination of the TPR-response to tyramine in WKY was in part reversed by prior administration of nadolol, atenolol or ICI-118551, with a non-additive effect of β_1_- and β_2_AR blockade (Figure 6). Significant differences were not detected between the L-659,066 and the βAR antagonist + L-659,066 pre-treated groups in SHR (Figure 6). When the β_3+1L_AR antagonist SR59230A was combined with L-659,066, the TPR-response to tyramine was not altered in WKY, whereas the vasoconstriction developed more slowly than after L-659,066 alone in SHR (Figure 6).

### The influence of β_1−3_AR and α_2_AR on the tyramine-induced tachycardia

The tyramine-induced tachycardia was not influenced in either strain by inhibition of α_2_AR-G_i_-signaling by L-659,066 or PTX, or by inhibition of cAMP-degradation by milrinone or stimulation of β_3_AR with BRL37344, either alone or combined with L-659,066 (Figure 7). The only exception was milrinone + L-659,066 which halved the HR-response in SHR (*P* ≤ 0.028) (Figure 7). As after nadolol alone (Berg et al., 2010), nadolol + L-659,066 eliminated the tachycardia in both strains (Figure 8). ΔHR was clearly reduced after atenolol + L-659,066 and to some extent also after SR59230A + L-659,066 in both strains (Figure 8), not different from that previously observed after atenolol and SR59230A alone (Berg et al., 2010). Different from that previously documented for ICI-118551 alone, i.e., no effect on the HR-response to tyramine in WKY and a slightly reduced response in SHR (Berg et al., 2010), ICI-118551 + L-659,066 reduced the tachycardia in WKY, but slightly increased the response in SHR (Figure 8). The effect of these drugs on the HR-response to tyramine was not paralleled by similar changes in the plasma catecholamine concentrations.

## Discussion

The main results in the present study were: (1) The α_2_AR antagonist L-659,066 required the presence of β_2_AR activity to enhance tyramine-induced norepinephrine overflow in WKY but was independent of β_1_AR signaling. (2) α_2_AR-mediated auto inhibition of norepinephrine release in SHR and epinephrine secretion in both strains was opposed primarily by β_1+1L_AR but also by β_2_AR activity. (3) α_2_AR and β_1+2_AR reciprocally modulated the TPR-response to the released norepinephrine in WKY but not in SHR. (4) In the presence of L-659,066, β_3_AR agonist stimulated vasoconstriction in WKY and α_2_AR auto inhibition in SHR during tyramine-induced norepinephrine release.

The reduction in tyramine-induced norepinephrine overflow to plasma after the β_1_AR antagonist atenolol was eliminated by additional pre-treatment with L-659,066 in WKY. The same was observed with the β_3+1L_AR antagonist SR59230A, most likely due to its ability to inhibit β_1L_AR (Berg, 2014b) (Figure 1). L-659,066 therefore increased release independent of β_1+1L_AR activity. However, the reduction induced by β_2_AR blocking antagonist, i.e., nadolol or ICI-118551, was somewhat or not at all reversed by L-659,066, respectively. It therefore appeared that β_2_AR-induced stimulation of release was a required substrate for α_2_AR auto inhibition in WKY. Since L-659,066 alone increased the tyramine-induced norepinephrine overflow in WKY, the β_2_AR were evidently active in stimulating release. However, amplifying the response to β_2_AR-G_s_ signaling by inhibiting PDE3-induced cAMP degradation with milrinone, did not enhance the effect of L-659,066 in WKY. Also the G_i_ inhibitor PTX did not alter norepinephrine overflow. These results may suggest that α_2_AR-G_i_ inhibition of norepinephrine release was activated in balance with β_2_AR-G_s_ stimulation of release. This balance may possibly result from a cAMP-PKA-dependent switch in β_2_AR-signaling from G_s_ to G_i_ (Daaka et al., 1997) when cAMP reached a certain level.

In SHR, where L-659,066 did not increase tyramine-induced norepinephrine overflow, additional pre-treatment with β_1_- as well as β_2_AR blockade allowed L-659,066 to increase the plasma norepinephrine concentration. Also SR59230A + L-659,066 increased norepinephrine overflow, most likely due to inhibition of the release-stimulating β_1L_AR. Thus, both β_1 and 1L_AR and β_2_AR counter-acted α_2_AR-mediated inhibition of norepinephrine release in SHR, different from that in WKY. It therefore appeared that excessive β_1+2_AR activity interfered with the inhibitory effect of α_2_AR-G_i_ on adenylyl cyclase in this strain.

Surprisingly, L-659,066 combined with the PDE3 inhibitor milrinone doubled norepinephrine release in SHR, even though milrinone alone had no effect. Thus, accumulation of cAMP after preventing its degradation greatly enhanced release only when the inhibitory action of α_2_AR-G_i_ on adenylyl cyclase was prevented (Figure 1). The fact that this was observed in SHR only was compatible with the augmented βAR hampering of α_2_AR auto inhibition in this strain.

Augmented norepinephrine overflow was also observed in SHR when L-659,066 was combined with BRL37344. Since BRL37344 may also activate β_1+2_AR-G_s_ signaling (Cernecka et al., 2014), this observation may be explained by a direct stimulating effect on adenylyl cyclase and subsequent norepinephrine release. However, as will be discussed below, BRL37344 in the presence of L-659,066 induced vasoconstriction in WKY, compatible with a stimulating effect on the G_i_-coupled β_3_AR. The potentiating effect of BRL37344 on release in SHR may therefore also result from β_3_AR-G_i_ stimulation, strengthening that activated by α_2_AR (Figure 1).

The secretion of epinephrine was tonically down-regulated by G_i_ in both WKY and SHR, indicated by the 6–7 times increase in the plasma epinephrine concentration in PTX-treated rats. A similar increase was seen in rats without tyramine-stimulated norepinephrine release (Berg et al., 2012). Although the plasma epinephrine concentration after pre-treatment with L-659,066 was less than that after PTX, the difference was not statistically significant, suggesting α_2_AR auto-inhibition of epinephrine secretion to be an important although not the only regulator of G_i_ in these cells. The secretion of epinephrine was not reduced by milrinone or β_1/1L/2_AR blockade alone. It therefore appeared that α_2_AR tonically inhibited the adrenal secretion of epinephrine in both strains with little βAR influence. In spite of this, β_1/2_AR blockade in both strains and also β_1L_AR antagonist in SHR potentiated the effect of L-659,066 on epinephrine secretion, apparently with a greater effect of the β_1_- than the β_2_AR in both strains. Thus, different from that observed for norepinephrine release in WKY but similar to that in SHR, β_1_- and β_2_AR in both strains and also β_1L_AR in SHR opposed α_2_AR-mediated inhibition of the secretion of epinephrine. Like tyramine-stimulated norepinephrine release in SHR but not in WKY, the effect of L-659,066 on the secretion of epinephrine was further enhanced when combined with milrinone or BRL37344 in both strains, most likely through the same mechanisms as discussed above.

The same receptors which presynaptically control catecholamine release are also located postsynaptically and modulate vascular tension and heart rate. Tyramine induces a massive release of norepinephrine without the normal physiological termination of the response by synaptic norepinephrine re-uptake through NET. One may therefore expect the concentration of norepinephrine within the synapse to be more than sufficient to maximally stimulate the postsynaptic receptors in all groups even though norepinephrine release differed. Differences in the cardiovascular response were therefore likely to be primarily due to drug influence on the postsynaptic receptors rather than reflect differences in release due to drug effect on the presynaptic receptors. This conclusion was supported by the fact that the tyramine-induced tachycardia was clearly hampered by β_1_- and β_2_AR antagonists also in the presence of L-659,066 which greatly increased the level of circulating catecholamines. The effect of tyramine does not depend on neuronal action potentials and is therefore not directly influenced by differences in neuronal activity. Due to the anesthesia, the cardiovascular response to tyramine was not modified by activation of baroreflexes, demonstrated by that the HR-response to tyramine was not influenced by atropine (Berg and Jensen, 2013). Moreover, large changes in BP induced by bradykinin or phenylephrine had no effect on HR in similarly anesthetized rats of both strains (Bjørnstad-Østensen and Berg 1994; Berg et al., 2012). Norepinephrine release was also not much influenced by the ganglion blocker hexamethonium, but being a nicotine receptor antagonist; it clearly reduced epinephrine secretion in both strains (Berg, 2014a). The vasoconstrictory TPR-response to the tyramine-induced norepinephrine release was due to α_1_AR activation since it was totally abolished by prazosin (Berg et al., 2010). However, concomitant vascular α_2_AR-βAR-activation will modulate this response, and this modulation differed in the two strains. In WKY, the norepinephrine-induced vasoconstriction was totally eliminated by PTX and L-659,066, showing that α_2_AR-G_i_-signaling was a major preserver of the α_1_AR-mediated vasoconstriction in this strain. This support was due to that α_2_AR-G_i_-signaling opposed β_1_- and β_2_AR-mediated vasodilatation, indicated by that β_1_- or β_2_AR antagonist prevented in part the L-659,066-dependent elimination of TPR-response to tyramine in WKY. Furthermore, accumulation of cAMP after pre-treatment with milrinone clearly reduced the peak-response to tyramine, and, in addition, potentiated the effect of L-659,066, thus precipitating a vasodilatory response to tyramine in milrinone + L-659,066-treated WKY. SR59230A did not alter the TPR-response to tyramine in WKY (Berg, 2014b) or in L-659,066-treated WKY, showing that β_3/1L_AR were not active and did not influence this response. Thus, when α_2_AR-mediated inhibition of adenylyl cyclase was prevented by L-659,066, an increased β_1+2_AR-dependent vasodilatation in response to the released norepinephrine and/or epinephrine was allowed. When in addition the degradation of cAMP was blocked, this effect was further enhanced (Figure 2). Thus, α_2_AR-βAR-modulation of the α_1_AR-mediated vasoconstriction was clearly functional in WKY.

In SHR, PTX, and L-659,066 reduced the TPR-peak-response but not the later response to tyramine, but, different from that in WKY, the TPR-response was not eliminated, and was not enhanced by additional pre-treatment with β_1,1L,2_AR antagonist. The slight delay observed in the development of the TPR-response in SHR pre-treated with SR59230A + L-659,066, may possibly result from inhibition of β_3_AR-G_i_-signaling. Thus, under the present conditions, there was little interaction between α_2_AR and βAR in the control of vascular tension in SHR. In pathophysiological conditions, including hypertension, enhanced activation of the phospholipase C (PLC)–protein kinase C pathway may lead to inhibition of vasodilatory voltage-sensitive K^+^ channels (K_V_) (Ko et al., 2010) (Figure 2). The presence of such inhibition in SHR was in fact confirmed by that antagonists against α_1_AR or angiotensin AT_1_ and ET_A_ receptors, which all activate PLC, enhanced the acute vasoconstrictory TPR-response to the K_V_ inhibitor 4-aminopyridine in SHR but not WKY (Berg, 2003). Since cAMP-induced vasodilatation may be mediated through a protein kinase A (PKA)-dependent opening of K_V_ (Aiello et al., 1998), the PLC-dependent inhibition of K_V_ in SHR was therefore likely to interfere with the α_2_AR-G_i_/βAR-G_s_-cAMP control of vascular tension (Figure 2). A PLC-dependent inhibition of K_V_ in SHR may therefore explain the absence of α_2_AR- and βAR-modulation of the TPR-response to tyramine-stimulated norepinephrine release in this strain. However, when α_2_AR-G_i_-signaling was prevented by L-659,066, and, at the same time, cAMP-signaling was amplified by milrinone, cAMP-mediated vasodilatation dominated the vascular tension response also in SHR. Thus, milrinone + L-659,066 eliminated tyramine-induced vasoconstriction in SHR. However, the effect was still less than that in WKY where milrinone + L-659,066 precipitated a tyramine-induced vasodilatation.

When the β_3_AR were stimulated with the agonist BRL37344 in WKY, the inhibitory effect of L-659,066 on the TPR-response to tyramine was reversed, with a stronger effect as catecholamine release progressed. This effect of BRL37344 could not be explained by its weak β_1+2_AR agonistic effect (Dolan et al., 1994), and BRL37344 did not interact with the putative β_4_AR (Malinowska and Schlicker, 1997), later identified as the β_1L_AR (Granneman, 2001; Kaumann et al., 2001). Since the β_3_AR is more resistant to catecholamine-induced desensitization than β_1/2_AR in human tissue (Wallukat, 2002; Rouget et al., 2004), this subtype may play a more prominent role during prolonged, high levels of norepinephrine such as during the late part of the tyramine-infusion period, particularly when combined with selective agonist. This vasoconstrictory component was likely to be mediated through β_3_AR-G_i_ signaling.

The tyramine-induced tachycardia was reduced after β_1_-, β_2_-, and β_1L(3)_AR antagonist in WKY also in the presence of L-659,066, apparently due to inhibition of postsynaptic βAR, independent of changes in norepinephrine release. In SHR, the tachycardia was reduced after L-659,066, halved after milrinone + L-659,066 and slightly increased after ICI-118551 + L-659,066. The reason for these changes was not obvious, but may result from receptor desensitization in the two former groups, and a possible switch from G_s_ to G_i_ for the β_2_AR (Daaka et al., 1997) in the latter group.

## Conclusions

α_2_AR-mediated inhibition of norepinephrine release required the presence of β_2_AR in WKY, but was independent of β_1_AR activity. The balanced α_2_AR-β_2_AR interaction in WKY may function to prevent excessive norepinephrine release during physiological conditions with increased epinephrine secretion such as hypoglycemia and exercise, since epinephrine is a better agonist for the β_2_AR subtype than norepinephrine. In SHR, α_2_AR inhibition of norepinephrine release was counter-acted by β_1_AR and β_2_AR activity, with an apparently stronger effect of the former. Although an α_2_AR-G_i_ tonic inhibition dominated the control of epinephrine secretion in both strains, their function was counter-acted by β_2_AR and even more by β_1_AR in both strains. The more prominent role of β_1_AR in counter-acting α_2_AR auto inhibition of catecholamine release in SHR may explain why β_1_AR blockers are useful as antihypertensive medication and protective in myocardial infarction and heart failure. The α_1_AR-mediated, vasoconstrictory TPR-response during tyramine-stimulated norepinephrine release was modulated by α_2_AR and β_1/2_AR in WKY. The latter interaction was not functional in SHR, most likely due to a PLC-dependent, reduced K_V_ vasodilatory influence on VSMC tension, a substrate for cAMP-induced vasodilatation.

### Conflict of interest statement

The author declares that the research was conducted in the absence of any commercial or financial relationships that could be construed as a potential conflict of interest.
